# Right renal cyst-duodenal fistula: an unusual complication of duodenal ulcer

**DOI:** 10.1016/j.eucr.2025.103255

**Published:** 2025-10-25

**Authors:** Qin-Wen Liu, Yong Cheng, Ge Li, Rui Jiang

**Affiliations:** Department of Urology, The Affiliated Hospital of Southwest Medical University, Luzhou, Sichuan, 646000, China

**Keywords:** Duodenorenal fistula, Duodenal ulcer, Renal cyst, Duodenal fistula

## Abstract

A 79-year-old female patient was admitted to the hospital with a one-month history of dull pain in the right upper abdomen, accompanied by fever, nausea, vomiting, melena and irritative urinary symptoms. Abdominal contrast-enhanced CT revealed a 5.8 × 5.0 cm cystic lesion in the right kidney with an air-fluid level. Upper gastrointestinal endoscopy detected a duodenal bulb ulcer accompanied by fistula formation.

**What would you do next?**

A) Endoscopic closure of duodenal perforation.
B) Fistula resection and perforated duodenal ulcer repair.
C) Nasojejunal tube implantation under gastroduodenoscopy.
D) Percutaneous puncture of the right renal abscess.

## 1 Introduction

Duodenorenal fistula is a rare complication, typically of chronic renal infection or perforated ulcer. Diagnosis is challenging due to non-specific symptoms. Surgical management, while common, carries high risks for elderly comorbid patients. We present a case of pyogenic right renal cyst and fistula secondary to duodenal ulcer, successfully managed with conservative endoscopic jejunal tube drainage, offering a less invasive alternative.

## 2 Case presentation

**Fig. 1:**
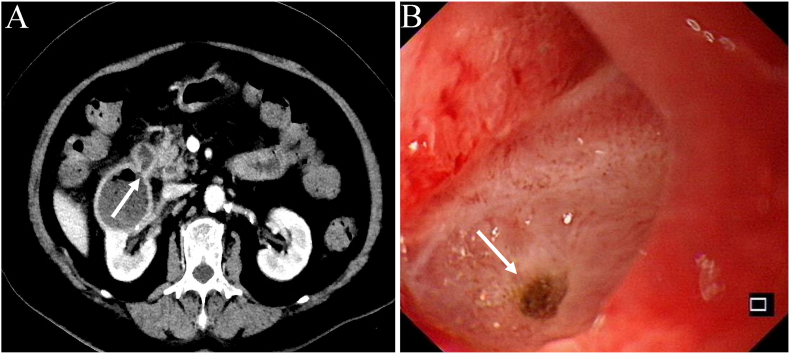
(A) An axial contrast-enhanced CT scan revealed the presence of an air-fluid level within the right renal cyst, characterized by a fistulous tract forming a connection with the duodenum (white arrows). (B) Upper gastrointestinal endoscopy demonstrated a circular ulcer located within the duodenal bulb (white arrows).

A 79-year-old female patient was admitted to the hospital with a one-month history of dull pain in the right upper abdomen, accompanied by fever, nausea, vomiting, melena, and irritative urinary symptoms. Her medical history included hypertension, diabetes mellitus, chronic gastritis, and a right renal cyst. Laboratory examinations showed a positive result for the fecal occult blood test and a hemoglobin level of 79 g/L. Urinalysis indicated the presence of 2+ leukocytes and occult blood. The levels of gastrointestinal tumor markers were within the normal range. Abdominal contrast-enhanced CT revealed a 5.8 × 5.0 cm cystic lesion in the right kidney with an air-fluid level. A localized defect was observed in the cyst wall, which established a connection with the duodenum (Fig. 1A). Upper gastrointestinal endoscopy detected a duodenal bulb ulcer accompanied by fistula formation (Fig. 1B).

**Fig. 2:**
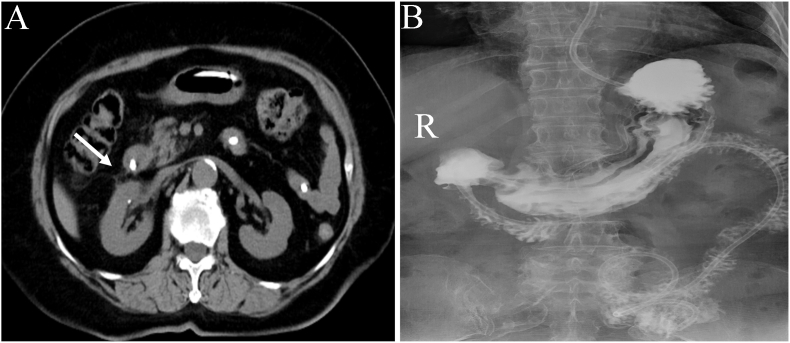
(A) A subsequent CT examination conducted one month later verified the complete resolution of the right renal cyst, as well as a clearly delineated boundary between the duodenum and the right renal periphery (white arrows). (B) The upper gastrointestinal series indicated no contrast extravasation from the duodenal bulb area one month later.

The patient underwent an upper gastrointestinal endoscopy for the insertion of a nasojejunal tube and subsequently received anti-infection, acid suppression, stomach protection, and enteral nutritional support. Within one week, there was a notable improvement in her symptoms, including abdominal pain and fever. At the one-month follow-up, the patient's hemoglobin level increased to 124 g/L. Abdominal CT indicated the complete resolution of the right renal cyst, along with a clear demarcation between the right kidney and the duodenal wall (Fig. 2A). An upper gastrointestinal series showed no contrast extravasation from the duodenal bulb (Fig. 2B). Subsequently, the nasojejunal tube was removed, and the patient resumed a normal diet, attaining a favorable prognosis.

## 3 Discussion

Duodenorenal fistula is a rare and intricate clinical condition that most frequently emerges as a complication of various factors, such as infection, calculi, malignancy, iatrogenic trauma, or peptic ulcer disorder. Diagnostic methods encompass abdominal contrast-enhanced computed tomography (CT), gastrointestinal contrast examination, and gastrointestinal endoscopy. Treatment modalities include conservative management, endoscopic closure, nephrectomy, and intestinal repair.^1^, ^2^, ^3^ Nevertheless, surgical intervention exhibits a high degree of invasiveness and entails substantial risks for elderly patients afflicted with multiple comorbidities.^4^ Moreover, the mere application of endoscopic closure is insufficient, as it does not address the infected cystic fluid.^5^

## 4 Conclusion

This report represents the initial characterization of a pyogenic renal cyst following a spontaneous duodenal ulcer perforation. Successful treatment was accomplished via endoscopic jejunal tube placement, which guaranteed adequate drainage and consequently promoted fistula closure. Surgical intervention is employed when conservative management proves ineffective.

## CRediT authorship contribution statement

**Qin-Wen Liu:** Visualization, Writing – original draft. **Yong Cheng:** Resources. **Ge Li:** Writing – review & editing. **Rui Jiang:** Visualization, Writing – review & editing.
